# General practice pharmacists in Australia: A systematic review

**DOI:** 10.1371/journal.pone.0258674

**Published:** 2021-10-14

**Authors:** Thilini Sudeshika, Mark Naunton, Louise S. Deeks, Jackson Thomas, Gregory M. Peterson, Sam Kosari

**Affiliations:** 1 Faculty of Health, Discipline of Pharmacy, University of Canberra, Bruce, Australian Capital Territory, Australia; 2 Faculty of Allied Health Sciences, Department of Pharmacy, University of Peradeniya, Peradeniya, Sri Lanka; 3 School of Pharmacy and Pharmacology, University of Tasmania, Hobart, Tasmania, Australia; International Medical University, MALAYSIA

## Abstract

**Background:**

The inclusion of pharmacists into general practices in Australia has expanded in recent years. This systematic review aimed to synthesise the literature of qualitative and quantitative studies, and identify the knowledge gaps, related to pharmacists working in general practice in Australia.

**Methods:**

This systematic review followed the Preferred Reporting Items for Systematic Reviews and Meta-Analyses guidelines. PubMed, EBSCOhost, EMBASE, and the Cochrane Library were searched from the inception of databases to January 2021. The search was focused on studies investigating general practice pharmacists in Australia. The quality of each study was appraised using the Mixed Method Appraisal Tool criteria. The narrative synthesis approach was utilised to describe data due to the heterogeneity among study designs and measures.

**Results:**

Twenty-five studies were included in this review. General practice pharmacists engaged in various non-dispensing patient care services, with medication management reviews being the primary activity reported. General practice pharmacists’ characteristics and an environment with a willingness of collaboration were the notable influencing factors for successfully including pharmacists in general practices. Factors that posed a challenge to the adoption of general practice pharmacists were lack of funding and other resources, poorly defined roles, and absence of mentoring/training.

**Conclusion:**

This review has summarised the characteristics, activities, benefits, barriers, and facilitators of including pharmacists in general practices in Australia. General practice pharmacists are well accepted by stakeholders, and they can engage in a range of patient-centred activities to benefit patients. There is a need for more robust research to explore the patient and economic outcomes related to clinical activities that a pharmacist can perform in general practice, as a foundation to developing an appropriate and sustainable funding model. The findings of this review will be beneficial for pharmacists, researchers, policymakers, and readers who wish to implement the role of general practice pharmacists in the future.

## Introduction

With the expansion of pharmacists’ roles, pharmacists have been included in primary healthcare teams to provide collaborative patient care in many countries. The co-location of pharmacists within primary healthcare teams has been studied in the United States of America (USA), United Kingdom (UK), Canada, Australia, The Netherlands, Ireland, Brazil, New Zealand, and Malaysia [1–8]. Studies from the USA, UK, and Canada have reported that the inclusion of pharmacists into primary healthcare teams has improved patients’ health outcomes and benefited patients in other ways [9–11].

The primary purpose of co-locating pharmacists in primary care teams is to improve medicines optimisation and patient safety [2,12,13]. However, the characteristics of models for including pharmacists into primary care vary according to the challenges and policies of healthcare systems in individual countries. For example, in the USA, the Patient-Centred Medical Home (PCMH) model was introduced to improve the quality of patient-centred services in primary healthcare and to lower healthcare costs [14]. The PCMH model relies on critical structural components, such as the shared use of health information, advanced performance indicators, trust and good rapport among providers and patients, and adequate financial reimbursement [15]. The PCMH model has offered numerous opportunities for pharmacists to contribute through diverse clinical activities to improve patient outcomes [10,16,17].

Canadians have investigated the inclusion of pharmacists into family practices since 2003. The “Integrating Family Medicine and Pharmacy to Advance Primary Care Therapeutics (IMPACT)” project was one of the largest trials launched in Ontario, Canada to investigate the inclusion of pharmacists in family health teams [18]. The main aim was to improve collaborative practice between pharmacists, family physicians, and allied health professionals in managing medication therapy [18–21]. In Canada, the public health system has provided funding for pharmacists in family health teams and pharmacists have rights to prescribe in some states/provinces [22,23]. Canadian studies have reported positively on patient-centred activities as a result of including pharmacists as a part of the primary care team [9,12,21]. Furthermore, there is evidence for continued expansion of pharmacists in family health teams across the country [20].

National Health Service England launched the Clinical Pharmacists in General Practice model in 2015 to help overcome the expanding workforce crisis of general practitioners (GPs) [11]. The over-arching purpose of including pharmacists into general practices across the UK is to improve patient care [13,24,25]. This includes providing extra help to manage chronic health conditions, education to those on multiple medicines, and better access to health assessments [25]. UK studies have shown that general practice pharmacists’ consultations can benefit patients [11,24–27]. There are currently over 1000 full-time equivalent clinical pharmacists working across general practices in the UK [28]. Like in Canada, pharmacists in general practices in the UK are funded by the government, and they have rights to prescribe [29]. The “Pharmacotherapy Optimisation through Integration of a Non-dispensing pharmacist in a primary care Team (POINT)” study in The Netherlands [5] and the “General Practice Pharmacist (GPP)” study in Ireland [4] were another two projects launched to explore the general practice pharmacist model.

In Australia, general practices are the frontline healthcare service that act as a gateway to specialist services [30]. The inclusion of pharmacists in Australian general practices has been evolving gradually over the last decade to provide non-dispensing services such as medication management services, medication safety initiatives, and patient education. The role of general practice pharmacists has been supported by the Australian Medical Association (AMA), Pharmaceutical Society of Australia (PSA), and various Primary Health Networks in Australia since 2015 [31]. However, Australia lags well behind other countries, such as the UK and Canada, in widening the role of general practice pharmacists across the country [32]. The reasons may be due to unique challenges and policies in the Australian healthcare system, including the lack of an appropriate funding model, limitation of prescribing rights in the scope of practice of pharmacists, and the absence of focusing on overall patient health outcomes when considering the remuneration [33]. Australia has a growing burden of chronic diseases, an increased ageing population, health workforce pressures, unacceptable inequities in health outcomes and access to services, and escalating healthcare expenditure [34,35]. It is still unknown whether there is any impact on these healthcare system challenges with the inclusion of pharmacists in Australian general practice.

There have been systematic reviews conducted globally to explore the effectiveness of the activities of pharmacists in general practices [36]; to investigate how the degree of inclusion of a non-dispensing pharmacist impacts medication-related health outcomes in primary care [37]; and to assess the competencies of general practice pharmacists [38]. However, to date, there has not been a review to assess the overall impact of the inclusion of pharmacists in Australian general practices. Even though there have been studies conducted in Australia related to general practice pharmacists [32,39–62], the literature has not been summarised to describe the role of general practice pharmacists and to identify knowledge deficits related to pharmacists working in general practice in Australia.

The aim of this systematic review was to synthesise the literature related to general practice pharmacists in Australia, and attempt to answer the following research questions:

1. What are the clinical and non-clinical activities being conducted by pharmacists in general practices in Australia?
2. What are the benefits/outcomes from the inclusion of pharmacists in the Australian general practice setting?
3. What are the perspectives of stakeholders, and the barriers and facilitators of the inclusion of pharmacists in the general practice setting in Australia?
4. What are the characteristics, qualifications, and experience of the pharmacists in general practices in Australia?
5. What are the knowledge deficits relating to pharmacists working in general practice in Australia?

## Methods

### Research design

A systematic review for mixed methodologies was followed, informed by the Preferred Reporting Items for Systematic Reviews and Meta-Analyses (PRISMA) systematic review guidelines and checklist (S1 Checklist) [63,64]. A mixed-methods systematic review methodology was employed due to the relative paucity of research on the general practice pharmacist model in Australia. This approach provided the flexibility required to incorporate various study methodologies and offered a systematic design to conduct the literature review to meet the aims of the research. The review was registered with the international database of prospectively registered systematic reviews (PROSPERO: CRD42019109963).

### Literature search strategy

A systematic literature search of PubMed, EBSCOhost, EMBASE, and the Cochrane Library was conducted from the inception of each database to January 2021. The search was focused on studies investigating general practice pharmacists in Australia. Full text peer-reviewed English language articles that involved qualitative, quantitative or mixed-method studies with any outcomes reported were included in the review. Search strategies for databases are provided in S1 Table. A manual search of the bibliography in the reference lists of identified articles was conducted and other related review articles were screened for additional relevant studies.

### Eligibility criteria

Articles were included in the review if the following conditions were met: (a) tested an intervention of pharmacists in general practice and/or obtained views of stakeholders (pharmacists, GPs, practice staff and patients) related to pharmacist services/activities in general practice; and (b) a pharmacist was co-located within a general practice clinic to provide non-dispensing medication-related activities.

Articles were excluded if any of the following conditions were met: (a) conducted in secondary, tertiary or other care settings (hospitals, nursing homes); (b) conducted outside of Australia; or (c) presented as editorials, protocols, letters, commentaries, and reviews.

### Data evaluation

Abstract and full-text screening was performed by two authors (TS, JT), who independently applied the eligibility criteria to 10% of the search results and checked inter-rater reliability. After finding 100% agreement, a single investigator (TS) rated the remaining 90% articles alone [65,66]. Full texts were reviewed where a decision could not be made on abstract and title alone. Title and abstract screening was conducted by using the software package Covidence for conducting systematic reviews [67]. Full-text screening involved using EndNote to manage and retrieve full-texts.

### Quality appraisal

The Mixed Methods Appraisal Tool (MMAT) was used to appraise the quality of empirical studies, as it covers a variety of methodologies [68]. The MMAT includes five core quality criteria for each of the following five categories of study designs: qualitative research, randomised controlled trials, non-randomised studies, quantitative descriptive studies, and mixed-method studies [68]. Critical appraisal of methodological quality and risk of bias assessment of included papers were undertaken independently by two reviewers (TS, SK). A third reviewer (MN) was consulted in the case of disagreement without reaching consensus.

### Data analysis

A narrative synthesis approach was used to synthesise the findings of the included articles, due to the heterogeneity of studies in the review with a range of methodologies [69,70]. First, a preliminary synthesis was conducted to search studies, and present results in a tabular form. Then the results were discussed by two reviewers (TS, SK) and structured into themes. The studies included in the narrative synthesis were then summarised within a framework to capture the features of each study with reference to the focus of this review: clinical and non-clinical activities of general practice pharmacists; benefits of general practice pharmacists; perspectives of stakeholders about general practice pharmacists; barriers and facilitators for the implementation of general practice pharmacists; and characteristics and training requirements of general practice pharmacists. Original investigators of the studies were contacted to obtain additional research information related to the framework [71].

## Results

### Literature retrieval

**Fig 1:**
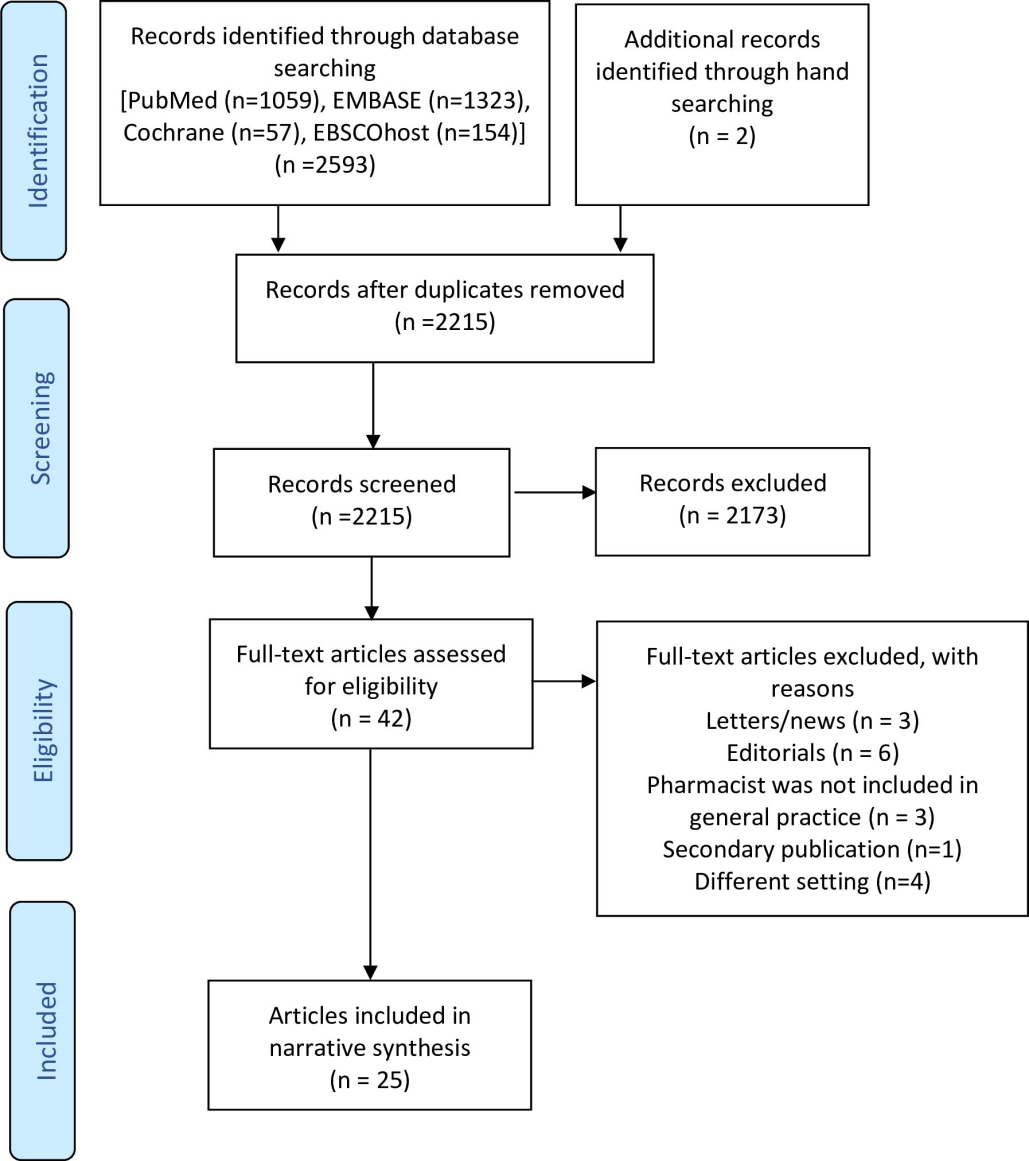
Flowchart depicting inclusion/exclusion.

A search of the databases identified 2215 records once duplicates were removed. Twenty-five articles met the full inclusion criteria and were included in this review [32,39–62]. Fig 1 shows the flow diagram of the study selection.

### Article characteristics

Characteristics of the 25 articles included in this review are provided in S2 Table. The included articles aimed to: (i) characterise the clinical and non-clinical activities that a pharmacist can perform in general practice [32,39,42,44,46,51–53,55,56,62], (ii) explore the pharmacist recommendations and general practitioner acceptance rates regarding medication-related decision-making in general practice [43,49–51], and (iii) explain the stakeholders’ perspectives, barriers and facilitators of the inclusion of a pharmacist into general practice [40,41,45,47,48,51,54,57–59,61]. The included articles utilised designs that were qualitative (40%, n = 10) [40,42,45,47,48,55,58,59,61,62], quantitative non-randomised control (32%, n = 8) [39,43,46,49,50,52,56,60], quantitative descriptive (8%, n = 2) [32,41], and mixed-method (20%, n = 5) [44,51,53,54,57].

### Risk of bias and quality assessment

All the included articles met the following screening criteria of MMAT: having clear research questions and addressing the research questions based on the collected data [65]. Almost all articles (92%, n = 23) were rated average or above average quality (S3 Table). Only 5 articles that utilised a qualitative design fulfilled all the criteria in the MMAT [44,46,47,58,60]. The articles which utilised a quantitative non-RCT design (32%, n = 8) or a mixed-method design (20%, n = 5) fulfilled 2–4 criteria [39,43,44,46,49–54,56,57,60], and quantitative descriptive design (8%, n = 2) fulfilled 3–4 criteria in the MMAT [31,40]. Overall, five articles (20%) fulfilled all criteria [44,46,47,59,61], eight articles (32%) fulfilled 4 criteria [32,40,42,46,49,50,54,62], ten articles (40%) fulfilled 3 criteria [39,41,52,52,54–58,60], and two articles (8%) fulfilled 2 criteria [42,43] for their particular research design using the MMAT. Details of the quality appraisal of the included articles are provided in S3 Table.

All the articles which utilised a qualitative design (n = 10, 40%) fulfilled 3 criteria or more in the MMAT. Among the ten qualitative studies, five studies used semi-structured interviews [45,46,58,59,61], two studies used a combination of semi‐structured and focus group interviews [40,47], two were case studies [55,62], and one study used an ethnographic approach [42]. Thematic analysis (6 out of 10, 60%) was the most widely used qualitative analysis method [45,47,48,58,59,61]. In the majority of non-randomised quantitative components and mixed-method studies, confounders and inconsistencies were not accounted for in the design and analysis. Small sample size/low response rate, lack of a control group, non-probability nature of sampling and researcher bias were the most notable limitations in the included articles [32,39–44,46,49–58,60,62].

### Synthesis of results

#### Clinical/Non-clinical activities conducted by the pharmacists in general practices and outcomes

The general practice pharmacist role comprised a variety of activities including medication reviews and reconciliation; clinical audits; adherence counselling; patient education on medical conditions and medications; reviewing and ordering of laboratory tests; healthy lifestyle advice; chronic disease management; medication information provision; and administrative work [32,40,42,44,46,48–53,55–57]. Medication management reviews performed by general practice pharmacists has been the leading clinical activity investigated in most articles, reflecting its role as the primary source of funding for Australian general practice pharmacists at present [32,39,43,46,49–51,62].

The inclusion of pharmacists into general practices can facilitate the delivery of multiple patient-centred activities to manage chronic diseases; however, there were only five studies demonstrating clinical outcomes for patients [44,53,55,56,62]. Two articles reported general practice pharmacists’ activities related to osteoporosis and asthma management in general practice [44,53]. Pharmacist-led consultations made significant improvements to the prescription of anti-osteoporosis medicines, vitamin D and calcium supplements in general practice [44]. A pilot study demonstrated the feasibility, acceptability, and benefits of a general practice pharmacist in asthma management [53]. This study showed that general practice pharmacists engaged in the following activities to manage asthma: performing asthma control assessment, making recommendations to adjust medication or device, providing education on correct device use and avoidance of trigger factors, and developing an asthma action plan [53]. Case studies and qualitative data from the study indicated potential hospital admission avoidance and stakeholder acceptability of asthma management by a general practice pharmacist [53]. Two articles reported the perspectives of community pharmacists and GPs on collaborative asthma management with general practice pharmacists [58,59]. The articles indicated that community pharmacists and GPs supported collaborative asthma management with general practice pharmacists [58,59]. Furthermore, these two articles illustrated the challenges in asthma management, barriers and facilitators for collaborative asthma management, and possible collaborative asthma care models with general practice pharmacists [58,59].

Three separate studies reported smoking cessation sessions, deprescribing in older persons, and reviewing medications in Human Immunodeficiency Virus (HIV)-infected patients utilising general practice pharmacists [55,56,62]. A pilot study reported that there was a smoking self-reported abstinence rate of 30% (20/66) and a verified abstinence rate of 20% (13/66) after at least 6 months, due to smoking cessation sessions conducted by a general practice pharmacist [56]. A case study report showed that deprescribing in older patients by general practice pharmacists could increase medication safety of elderly patients in general practices [55]. One case study demonstrated that review of medications by a general practice pharmacist could improve patient outcomes and prevent long-term consequences in patients with HIV [62]. Further, several studies have described other potential roles for general practice pharmacists: prescribing, managing insomnia, providing specialised medication information to GPs, screening for undiagnosed diseases, on-site dispensing, and optimising medication records [32,40–42,48,51,52,57,61].

#### Benefits of pharmacists in general practices

The inclusion of pharmacists in general practices can increase communication and collaboration between pharmacists and GPs [32,40,41,47,48,51,52,54,57,58]. The included articles reported that collaborative interventions by general practice pharmacists can identify and resolve medication-related problems [43,46,50,52,55,62]. For example, Benson et al in 2018 reported that general practice pharmacists identified 1124 medication-related problems from 15 general practices in Western Sydney over six months [50]. Tan et al reported that the median number of medication-related problems per patient identified by a general practice pharmacist at baseline was 2, decreasing to zero at six months after general practice pharmacists’ interventions [46]. Two studies have reported that general practice pharmacists’ consultations can improve the medication adherence of patients in general practices [46,49]. One study indicated that general practice pharmacist’s consultations improved adherence as rated by the Morisky Scale (44.1% to 62.7%, p = 0.023) and Tools for Adherence and Behaviour Screening (23.6% to 57.6%, p = 0.019) in patients who had one or more risk factors for medication-related problems [46].

It has been shown that a general practice pharmacist is effective at improving the timeliness of completion of Home Medicine Reviews (HMR) [39]. A complete HMR service includes the service provided by a GP, an accredited pharmacist (a pharmacist who is licensed to conduct medication management reviews, including HMRs and Residential Medicine Management Reviews (RMMR)), and input from a multidisciplinary team from the time the patient is identified through to the implementation and ongoing monitoring of the medication management plan and any follow-up service(s) as required [72]. In 2012, Freeman et al reported that a general practice pharmacist completed HMRs in a shorter time (within 20 days) than an accredited pharmacist who is external to general practice (within 56 days) [39]. The same study showed that the number of incomplete medication reviews that were not received by the medical centre, and HMR reports not reviewed or billed by GPs were significantly reduced from 56% to 6% after including a pharmacist into general practice [39]. Furthermore, this study demonstrated potential savings of AUD 17,374 per year for general practices from the completion of HMRs by a general practice pharmacist [39]. Kosari et al in 2020 reported that activities conducted by two general practice pharmacists saved time for GPs, potentially allowing for additional GP-patient consultations [60]. In this study, the time saved for GPs was estimated to be 23.9 hours per month for a full-time pharmacist working 37.5 hours per week [60]. This study reported that two part-time general practice pharmacists’ activities generated income of AUD 7000 for general practices over 19 weeks [60]. Furthermore, this study compared the cost of employing pharmacists against the income generated by them and reported that general practice pharmacists generated income between AUD 0.61–1.20 per AUD 1.00 of a pharmacist’s salary [60].

Several articles have shown that recommendations made by general practice pharmacists to GPs were frequently accepted (mean acceptance rate: 79.8±10.7%) [39,43,49,50]. It has been shown that general practice pharmacists’ involvement in clinical activities to support GPs increased with time working at the practice [52]. It was found that the addition of a general practice pharmacist improved liaison between the general practice and outreach services in two articles [57,58]. One article highlighted that the practice pharmacist’s role was intended to be complementary to and not competitive with that of community pharmacists [58], while the article by Baker et al highlighted that general practice pharmacists liaised more with hospitals and other outreach services than accredited pharmacists who worked in conjunction with GPs [57].

Included articles in this review have not stated any clinical disadvantages of the inclusion of general practice pharmacists. However, one article reported that the role of community pharmacists may be duplicated in general practice for smoking cessation and inhaler device education, with a subsequent loss of income for the community pharmacy [54]. Overall, the reported benefits of having a general practice pharmacist are summarised in Table 1.

**Table 1:** Benefits of the inclusion of general practice pharmacists.

Benefits
Increase communication and collaboration with GPs [32,40,41,47,48,51,52,54,57,58]
Identify and resolve medication-related problems [43,46,50,52,55,62]
Improve medication adherence [46,49]
Reduce time to complete a medication review [39]
Potential savings in money and time [39,60]
Increase the acceptance of pharmacists’ recommendations by GPs [39,43,49,50]
Improve patient health outcomes [40,48,57,62]
Act as a conduit between the patient’s general practice and community pharmacies, hospitals, and other outreach services [57,58]

*GP-General Practitioner.

#### Perspectives of stakeholders, barriers, and facilitators of the inclusion of pharmacists into general practices

There were eleven articles (44%) exploring the perspectives of stakeholders about including pharmacists in general practices [40,41,45,47,48,51,54,57–59,61]. The studies have indicated that general practice pharmacists were highly accepted into the general practices by stakeholders, including GPs, pharmacists (accredited and community pharmacists), patients, nurses, and practice managers. The main themes identified by the stakeholders were “benefits of general practice pharmacists”, “barriers and facilitators of the inclusion of general practice pharmacists”, “pharmacists’ attributes”, “collaboration”, “logistical challenges” and “sustainability” [40,41,45,47,48,51,54,57–59,61].

*Barriers*. There was a number of barriers identified for the inclusion of pharmacists into general practices in Australia. The main barrier for including pharmacists into general practices was funding/lack of government support [40,41,45,47,48,51,54,57–59,61]. Logistical challenges and role clarity were other general impediments identified by the pharmacists and GPs [40,41,45,47,48,51,54,57–59,61]. A lack of clinic space, lack of time for staff to engage with the practice pharmacist, and lack of patient time to attend multiple visits were the main logistical challenges described in the articles [40,47,51,57,59,61].

Medical culture, attitudes and perception of general practice staff and patients were mentioned as barriers to the implementation of general practice pharmacists in six articles [40,45,47,51,58,61]. Underutilisation of general practice pharmacists due to poor awareness of general practice staff towards the potential services that a practice pharmacist could offer was discussed in three articles [51,54,57]. The pharmacists have reported that absence of mentors to guide them in their roles and absence of programs to train them as a general practice pharmacist were barriers for implementing this practice model [51,54,59,61].

*Facilitators*. Stakeholders emphasised the GPs’ willingness for collaboration, rapport, communication and team support as the main facilitators for the inclusion of pharmacists in general practices [40,41,45,47,49,51,54,57,58]. Pharmacists’ characteristics including proactivity, good communication skills, clinical competency, credibility and adaptability were other facilitators identified in this review [45,47,51,54,57]. The benefit for patients through general practice pharmacists’ services, such as providing education, was an influencing factor reported by several articles [40,48,57,59,61]. Only two articles reported having a clearly defined scope of practice as a factor facilitating the inclusion of general practice pharmacists [40,57]. GPs who actively recommended the pharmacist to the patient were stated as important to facilitate the successful implementation of general practice pharmacists [51]. Not surprisingly, remuneration/external funding was another factor that can facilitate the inclusion of general practice pharmacists [40,45,61]. A study by Freeman et al in 2012 reported that stakeholders thought that a mix of government and private funding was an appropriate model of remuneration for pharmacists in general practice [40]. The barriers and facilitators for the inclusion of general practice pharmacists are summarised in Table 2.

**Table 2:** Barriers and facilitators for the inclusion of general practice pharmacists in Australia.

Barriers	Facilitators
Funding/lack of government support [40,41,45,47,48,51,54,57–59,61]	Willingness of collaboration (rapport, communication, and team support) [40,41,45,47,49,51,54,57,58]
Role clarity [40,41,45,47,48,51,54,57–59]	
Logistical challenges (office space, lack of resources and time) [40,47,51,57,59,61]	Pharmacists’ characteristics [45,47,51,54,57]
Lack of awareness of pharmacists’ activities and underutilisation [51,54,57]	Clearly defined scope of practice [40,57]
	Benefits for patients and better patient outcomes [40,48,57,59,61]
Medical culture, perception, and attitudes [40,45,47,51,58,61]	Recommendation by GPs to patients [51]
Lack of mentors [51,54,59,61]	Remuneration/external funding [40,45,61]

*GP-General Practitioner.

#### Characteristics, qualifications, and experience of pharmacists in general practice

Thirteen articles (52%) included in this review mentioned the general practice pharmacists’ characteristics, qualifications, and experience [32,40,42,43,45–47,49,52,54,56,57,60]. In eleven articles, the pharmacists recruited had accreditation to perform medication reviews [32,39,40,43,45–47,49,54,57,60]. Freeman et al in 2014 conducted a survey to explore the characteristics of general practice pharmacists Australia-wide and reported that 85% (n = 22) of general practice pharmacists were accredited to conduct medication management reviews, with 27% (n = 7) having been accredited for 13–15 years, 23% (n = 6) accredited for 1–3 years, and 19% (n = 5) accredited for 7–9 years [32]. General practice pharmacists’ experience varied in the included articles in this review. Two articles reported pharmacists’ experience ranged from 3–31 years [52,54], one article reported that pharmacists’ experience ranged from 3–14 years [60], two articles reported that pharmacists had experience of more than 8 years [46,47], and one article reported that pharmacists had experience of more than 10 years [49].

Only three articles included in this review reported the educational qualifications of general practice pharmacists [32,49,57]. One of these articles reported that general practice pharmacists had postgraduate qualifications related to clinical pharmacy (clinical pharmacy is a field in which pharmacists provide direct patient care to optimise medication use, often in the hospital setting) [49]. One quantitative descriptive study conducted Australia-wide reported that 58% (n = 15) of general practice pharmacists had postgraduate qualifications: coursework masters (27% (n = 7)), graduate diploma (23% (n = 6)) or a research doctorate (15% (n = 4)) [32]. Furthermore, one article reported accredited pharmacists’ opinions about desirable characteristics of pharmacists (qualifications, skills, experience, training) to work effectively in general practice: prior experience as a hospital pharmacist, communication skills, problem solving skills, Information Technology (IT) skills and understanding pharmacoeconomics [57]. The reported characteristics, qualifications, experience, skills, and desirable special training of general practice pharmacists are summarised in Table 3.

**Table 3:** Overview of general practice pharmacists’ characteristics described in the articles.

Main characteristic/ requirement	Results/description
Accreditation	Accreditation to perform medication management reviews [32,39,40,43,45–47,49,54,57,60]
Experience	Minimum 3 years of experience [46,47,49,52,54,60], prior hospital experience (desirable) [57]
Educational qualifications	Postgraduate qualifications [32,49,57]
Special training	Desirable training: Introductory modules by PSA, GP software, spirometry, mental health, online asthma counselling, and motivational interviewing and health coaching [57] One article stated that pharmacist had completed training related to smoking cessation [56]
Skills	Communication skills, problem solving skills, IT skills, understanding pharmacoeconomics [57]

*PSA-Pharmaceutical Society of Australia, GP-General Practitioner, IT-Information Technology.

## Discussion

Over the past 20 years, the role of general practice pharmacists has been expanding worldwide. This review included articles with various methodologies, which explored factors related to the general practice pharmacist model in Australia. It adds a valuable insight on the overall view of pharmacists being embedded into general practices in Australia over the last decade.

### Benefits and activities of general practice pharmacists

Overall, our findings indicated that the inclusion of pharmacists in Australian general practices benefited patients by improving medication safety, mainly through resolving medication-related problems [40,43,46,48–50,52,55,57,62]. Increasing communication between general practice pharmacists and GPs is critical in order to facilitate the effective functioning of primary care services in the context of growing demand [73]. Our findings indicated that the inclusion of pharmacists in general practices can increase communication with general practice team members [32,40,41,47,48,51,52,54,57,58]. Moreover, general practice pharmacists’ recommendations were highly accepted by GPs, which demonstrates rapport between these two professionals [39,43,49,50]. These findings are supported by international studies conducted in the UK and Canada [9,13,20,25].

There are mixed views on the liaison between community pharmacists and general practice pharmacists. In this review, it was shown that a key priority of the general practice pharmacist was to act as a conduit between community pharmacy, hospital pharmacy, and the general practice [57,58]. Community pharmacists appeared less enthusiastic towards pharmacists working in general practice according to one Australian article [54], while international studies have reported mixed views on the relationship between community pharmacists and general practice pharmacists: this includes a good rapport, and significant tensions stemming from professional hierarchy and competing business-related interests [9,74,75]. However, there is a knowledge gap in examining the impact of liaison between community pharmacists and general practice-based pharmacists, mainly around HMRs, smoking cessation, and lifestyle advice [54,76]. Therefore, further studies are required to explore collaboration between community pharmacists and general practice pharmacists.

The general practice pharmacist can engage in various activities, including medication safety initiatives such as clinical audits, medication management, education, and administrative work to improve patient health outcomes in general practices [32,40,42,44,46,48–53,55–57,62]. However, the primary function of general practice pharmacists reported by the majority of the articles was medication management reviews, in line with the stakeholders’ desired function of practice pharmacists [32,39,43,46,49–51,62]. This result is expected because HMRs and RMMRs are the only government-remunerated cognitive services that a pharmacist can conduct outside the community pharmacy in Australia [77]. Even though these activities are consistent with the international literature, general practice pharmacists’ activities are relatively limited by the scope of practice in Australia [9,13,78]. In particular, Australian pharmacists are unable to prescribe like general practice pharmacists in the UK and Canada, and are therefore very reliant on GPs adopting their recommendations [11,23,43,39,43,49,50,79]. Positively, however, the general practice pharmacists’ recommendations were highly accepted by GPs [39,43,49,50].

The activities that could be performed by a general practice pharmacist may save the time of GPs and generate income for the practice. A systematic review has reported that advanced pharmacy services appear to be cost-effective when delivered in community and primary care settings, but not in domiciliary settings [80]. A recent Irish study has reported that activities (medication reviews and involvement in the repeat prescribing process) by three general practice pharmacists working for 10 hours per week over 26 weeks saved approximately EUR 57 000 per year for general practices [81]. In Australia, as elsewhere, there is limited data on cost-benefits of the role of general practice pharmacists. The potential time saved for GPs by tasks being undertaken by part-time general practice pharmacists in one study was estimated to be 23.9 hours per month for a full-time pharmacist working 37.5 hours per week [60]. Based on the findings of this review, it is recommended to further evaluate clinical and non-clinical activities, and the cost-benefits of general practice pharmacists, with robust methodologies.

### Barriers and facilitators of the inclusion of general practice pharmacists

The general practice pharmacist model has been well accepted by stakeholders [40,41,45,47,48,51,54,57–59,61]. This systematic review has highlighted several barriers, including lack of resources (funding/lack of government support, logistic challenges), lack of role definition, and lack of mentoring/training. Our results are broadly consistent with international studies regarding the barriers that pharmacists face when attempting to integrate into primary care teams [9,21,82,83]. Appropriate planning and preparation can be utilised to mitigate the identified barriers [82]. Identified factors that enhanced the inclusion of general practice pharmacists were willingness to collaborate, team support, pharmacists’ characteristics, recommendation by GPs to patients, a clearly defined scope of practice and proven benefits for patients [40,41,45,47,48,51,54,57–59,61]. Positive inclusion of pharmacists into general practices appeared to be greatly reliant on the individual characteristics of the pharmacists. Pharmacists who were pro-active, adaptive, and confident were more likely able to facilitate their inclusion in Australian general practices. These findings are compatible with studies conducted in Canada [9,21,82].

This review has revealed that there is sufficient evidence to explain barriers and facilitators for the inclusion of Australian general practice pharmacists [40,41,45,47,48,51,54,57–59,61]. Unfortunately, these barriers are persisting, and it suggests that actions made to minimise barriers are still lacking. The findings of the review provide a better understanding of the barriers and facilitators when developing strategies to ensure pharmacists are successfully included in general practice teams.

### Qualifications and training of general practice pharmacists

Accreditation to perform medication reviews, postgraduate qualifications, experience as a registered pharmacist, and specific skills such as communication and analytical skills, and training were the main desirable characteristics of general practice pharmacists in the articles included in this review [32,40,42,43,45–47,49,52,54,56,57,60]. The general practice pharmacists in Australia have not received formal training or guidelines before their employment. This contrasts with the inclusion of clinical pharmacists in general practices in the UK and Canada. In the UK, a government-funded general practice pharmacist training pathway was introduced for pharmacists employed in general practice. This consisted of an 18-month mandatory training program which provided a combination of study days, peer-learning groups, assessments, and access to three support functions—an education supervisor (offering individualised educational support), a GP clinical supervisor (based in practice, offering day-to-day clinical support), and a clinical mentor (an experienced clinical pharmacist) [10]. In Canada, the IMPACT training session was introduced as a transitional programme for pharmacists entering family practice to provide guidance and strategies. In addition, the “Adapting pharmacists’ skills and Approaches to maximize Patient’s drug Therapy effectiveness” (ADAPT) educational program was developed to provide a standard approach to medication assessment, team collaboration, patient assessment, evidence-based decision making and documentation, facilitated through an e-learning program for pharmacists in family health teams [20,84]. Similar models are desperately needed in Australia. Recently, the PSA has developed introductory modules for pharmacists wishing to work in general practices, focusing on the practical aspects of working as a general practice pharmacist [85]. However, the PSA training modules only provide an overview of the core knowledge and skills required by general practice pharmacists.

A Delphi study has reported 26 general practice pharmacist activities as educational needs in Australia, which was consistent with the themes in international studies [86]. This study has highlighted those activities related to medication management, chronic disease management, research, patient examination and screening, and audit and quality assurance were the major educational needs of general practice pharmacists [86]. By defining the core needs of the role, appropriate training and education programs can be developed for general practice pharmacists. Our findings support that comprehensive training programs are required for general practice pharmacists in Australia, as identified by Benson et al in [86].

### Knowledge gap

Whilst the literature to date demonstrates several studies exploring the general practice pharmacist model in Australia, there is still limited evidence regarding certain areas. These include: awareness of stakeholders towards the activities that general practice pharmacists could perform, cost-benefits of including general practice pharmacists, interprofessional collaboration after including pharmacists into general practice teams, training and education needs of general practice pharmacists, guidelines/recommendations to utilise pharmacists in the general practice setting, and liaison of general practice pharmacists with community pharmacists and other out-reach services such as hospital pharmacies and aged-care facilities. While a limited number of studies indicated that general practice pharmacists’ activities improved patients’ health outcomes, evidence based on the use of robust methodologies is still lacking. Only one recent study, published outside of the search period of this review, utilised a randomised controlled trial [87]. This article reported that general practice pharmacists’ medication management services reduced the number of unplanned hospital admissions for patients recently discharged from hospitals who were prescribed five or more long-term medicines or had a primary discharge diagnosis of congestive heart failure or exacerbation of chronic obstructive pulmonary disease [87]. The estimated incremental net cost-benefit of the general practice pharmacist-led medication management services was AUD 5072 per patient, with a benefit-cost ratio of 31:1 [87]. As such, there is a need for more randomised controlled trials to explore the patient outcomes related to clinical activities that a pharmacist can perform in general practice.

### Strengths and limitations

A strength of this systematic review was the inclusion of studies with various methodologies, including quantitative, qualitative and mixed-methods studies. This allowed a comprehensive and robust characterisation and evaluation of aspects of the implementation of general practice pharmacists in Australia. Moreover, abstract screening and data extraction were completed through compliance with best practice. Another strength of this review was contacting original investigators to obtain additional research information to improve the accuracy of the review.

The study has several limitations that constrain our conclusions. First, this review found a relatively small number of relevant studies. Only 25 studies were identified with various methodologies [32,39–62], which led to the choice of a narrative synthesis approach for data analysis. Second, there is the potential of researcher bias in the evaluation, although two reviewers independently assessed the articles. Third, the role of general practice pharmacists is relatively new in Australia; studies have been conducted in a limited number of general practices that believed in value of the inclusion of pharmacists in general practice. Therefore, the findings related to clinical disadvantages of the inclusion of general practice pharmacists may not be reported in the included articles in this review and the findings may not be generalisable to all Australian general practices if this model is rolled out in the future.

### Study implications

The study has several implications. There is a need to:

- Utilise robust methodologies to assess clinical/non-clinical activities of general practice pharmacists.
- Act on the barriers and facilitators for the successful implementation of general practice pharmacists, especially those relating to funding and training.
- Explore further in the areas where there are knowledge gaps related to the general practice pharmacist model identified through this review.

The findings of this systematic review will be useful for researchers, policy makers, and stakeholders to strengthen the role of general practice pharmacists in Australia. Furthermore, our findings will be beneficial for readers who wish to implement a team-based primary care model in their countries where the inclusion of pharmacists into primary care teams is not established yet.

## Conclusion

This systematic review has summarised the characteristics, activities, benefits, barriers, and areas requiring further exploration relating to pharmacists working in general practice in Australia. General practice pharmacists are well accepted by stakeholders; they can engage in a range of clinical/non-clinical activities to benefit patients and general practices. This review has revealed that more actions are required on the factors that posed a challenge to the inclusion of pharmacists in general practice. Furthermore, this review has suggested more robust research to explore the patient and economic outcomes related to clinical activities that a pharmacist can perform in general practice, as a foundation to developing an appropriate and sustainable funding model.

## Supporting information

S1 Checklist Preferred reporting items for systematic reviews and meta-analyses (PRISMA) checklist. (DOCX)

S1 Table Electronic database search strategy. (DOCX)

S2 Table Characteristics of the articles included in the review. (DOCX)

S3 Table Quality appraisal of the articles included in the review. (DOCX)
