# Supplementary material for: General practice pharmacists in Australia: A systematic review
Source: PLoS One. 2021 Oct 14;16(10):e0258674. doi: 10.1371/journal.pone.0258674 (PMC8516208; doi:10.1371/journal.pone.0258674)
Supplement: S1 Table — (DOCX) [file pone.0258674.s002.docx]

**S1 Table: Electronic database search strategy**

| **Database** | **Search terms** |
| --- | --- |
| EBSCOhost | (Pharmacists OR Pharmacist OR “Pharmaceutical services” [Subject Terms] OR “Pharmacy services” [Subject Terms]) AND (General practice [All text] OR “Medical practice” [Subject Terms] OR "Primary care" [Subject Terms] OR "Primary healthcare" [Subject Terms]) AND Australia [All text]  Limit: English, Full text articles |
| Cochrane Library | (Pharmacists OR Pharmacist OR “Pharmaceutical services” [Title, abstract, keywords] OR “Pharmacy services” [Title, abstract, keywords]) AND (General practice [All text] OR “Medical practice” [Title, abstract, keywords] OR "Primary care" [Title, abstract, keywords] OR "Primary healthcare" [Title, abstract, keywords]) AND Australia [All text]  Limit: English, Full text articles, trials |
| PubMed | (Pharmacist OR Pharmacists OR Pharmacy Services OR Pharmaceutical Services) AND (General practice OR "Medical practice" OR "Primary care" OR "Primary healthcare") AND (Australia)  Limit: English, Full text articles |
| EMBASE | (Pharmacists OR Pharmacist OR “Pharmaceutical services” [Title, abstract, keywords] OR “Pharmacy services” [Title, abstract, keywords]) AND (General practice [All text] OR “Medical practice” [Title, abstract, keywords] OR "Primary care" [Title, abstract, keywords] OR "Primary healthcare" [Title, abstract, keywords]) AND Australia [All text]  Limit: English, Full text articles, Australia |
