# Supplementary material for: General practice pharmacists in Australia: A systematic review
Source: PLoS One. 2021 Oct 14;16(10):e0258674. doi: 10.1371/journal.pone.0258674 (PMC8516208; doi:10.1371/journal.pone.0258674)
Supplement: S2 Table — (DOCX) [file pone.0258674.s003.docx]

**S2 Table: Characteristics of the articles included in the review**

| **Primary author and year** | **Aim** | **Location** | **Study design** | **Description** | | | | **Outcome(s)** |
| --- | --- | --- | --- | --- | --- | --- | --- | --- |
|  |  |  |  | No. of general practices: pharmacists | Duration of the intervention | Pharmacist time spent in practice | Sample size/ participants |  |
| Freeman et al 2012 [39] | Describe the effect of integrating a pharmacist into the general practice team on the timeliness and completion of pharmacist conducted medication reviews | Brisbane, Queensland | Quantitative non-RCT/ pre -post intervention including a retrospective analysis | 1: 1 | 12 months | Part-time* | 70 patients referred for medication review in the pre- inclusion phase and 314 patients referred in the post-inclusion phase | The inclusion of a pharmacist into the general practice team was associated with an increase in the timeliness and completion rate of medication reviews. |
| Freeman et al 2012 [41] | Seek the views of pharmacists, GPs, and consumers on integrating pharmacists into general practice | Australia-wide | Quantitative descriptive | N/A | N/A | N/A | 1038 (829 pharmacists, 167 consumers and 42 GPs) | There was support from stakeholders for integrating pharmacists’ activities into general practices. |
| Freeman et al 2012 [40] | Describe the opinions of local stakeholders on the inclusion of a pharmacist into the Australian general practice environment | South-East Queensland | Qualitative/ content analysis | N/A | N/A | N/A | 58 (Interviews: 10 pharmacists, 4 GPs, 4 practice managers; focus groups: 18 pharmacists, 4 GPs, 18 consumers) | Identified stakeholders’ views on potential roles, benefits, barriers, facilitators, and remuneration methods of the inclusion of general practice pharmacists to develop a new model. |
| Freeman et al 2012 [42] | Provide an account of the activities carried out by a primary care practice pharmacist on a day-to-day basis | Brisbane, Queensland | Qualitative/ ethnographic approach | 1: 1 | 3 months | 3.5 days/ week (296 hrs) | 1 pharmacist reported activities | Identified a broad range of activities that a general practice pharmacist could perform in the primary care setting as part of a multidisciplinary team. |
| Freeman et al 2013 [43] | Compare medication review reports conducted by an integrated practice pharmacist with medication reviews conducted by pharmacists practicing externally to a medical centre, and reviews conducted in patients’ home | Brisbane, Queensland | Quantitative non-RCT/ pre post intervention including retrospective analysis | 1: 1 | 12 months | Part-time* | 14 (1 general practice pharmacist, 13 external pharmacists) | The general practice pharmacist identified a lower rate of drug-related problems than the external pharmacists due to the access to patients’ medical records.  The GPs implemented a higher percentage of recommendations made by general practice pharmacists (acceptance rate 71%). |
| Freeman et al 2014 [32] | Characterise pharmacists practising in the Australian general practice setting | Australia-wide | Quantitative descriptive | N/A | N/A | N/A | 26 general practice pharmacists | Identified characteristics of pharmacists working in the Australian general practice setting. |
| Tan et al 2014 [44] | Evaluate the impact of a drug use evaluation program on osteoporosis management in general practice | Melbourne, Victoria | Mixed- method | 2: 2 | 12 months | 8 hours/ week | 225 patients at the baseline audit, and 240 at the post-intervention audit (213 patients at both time points) | General practice pharmacists have produced significant improvements in osteoporosis management in general practice setting. |
| Tan et al 2014 [45] | Elicit the views of GPs and pharmacists on the inclusion of pharmacists into general practice in Australia to explore the proposed roles for a general practice pharmacist, and the factors influencing inclusion | Melbourne, Victoria | Qualitative/ thematic analysis | N/A | N/A | N/A | 27 (11 GPs and 16 pharmacists, including 2 GPs and 3 pharmacists who had previous experience with/as a general practice pharmacist) | The inclusion of pharmacists into general practices was well accepted by GPs and pharmacists.  Identified various inclusion methods, and barriers and facilitators for the inclusion of general practice pharmacists. |
| Tan et al 2014 [46] | Evaluate the effectiveness of consultations by pharmacists based within primary care medical practices | Melbourne, Victoria | Quantitative non- RCT/ pre post intervention study | 2: 2 | 6 months | 8 hours/ week | 82 patients were recruited and 62 completed the study | Medication-related problems were identified and resolved through general practice pharmacists’ consultations.  General practice pharmacists were associated with improving medication adherence in patients at risk of medication misadventure.  General practice pharmacist services were well accepted by patients and staff. |
| Tan et al 2013 [47] | Explore general practice staff, pharmacist, and patient experiences with pharmacist services in Australian general practice clinics | Melbourne, Victoria | Qualitative/ thematic analysis | 2: 2 | 6 months | 8 hours/ week | 34 (18 patients, 9 GPs, 4 practice nurses, 1 practice manager and 2 general practice pharmacists) | Stakeholders were receptive to integrate pharmacists into general practices. |
| Bajorek et al 2015 [48] | Explore the perspectives of GP super clinic staff on current and potential (future) pharmacist-led services provided in this setting | Blue Mountains, New South Wales | Qualitative/ thematic analysis | 1: 1 | Not described | 8 hours/ fortnight | 9 (3 GPs, 1 pharmacist, 1 nurse, 1 business manager and 3 reception staff) | The inclusion of pharmacists into a multidisciplinary super clinic could solve issues in medicines management.  The pharmacist in super clinic model was well accepted by stakeholders.  Identified the potential roles of the pharmacist when providing integrated care. |
| Benson et al 2018 [50] | Identify and classify the drug-related problems detected as a result of pharmacist activities within general practice settings.  Compare the number of pharmacist recommendations and GP acceptance rates as a result of pharmacist patient consultations across multiple general practice sites | Western Sydney, New South Wales | Quantitative non-RCT/ multi-centre prospective observational study | 15: 6 | 6 months | Not described | 493 patient consultations | General practice pharmacists were effective in identification of drug-related problems.  General practice pharmacists’ recommendations in managing drug-related problems were highly accepted by GPs (70%). |
| Benson et al 2018 [49] | Describe, classify, and analyse the recommendations made by pharmacists to GPs in a general practice setting | Western Sydney, New South Wales | Quantitative non-RCT/ multi-centre prospective observational study | 13: 4 | 6 months | One pharmacist: full-time, visited 9 practices/ week and 3 pharmacists: part-time, visited up to 2 practices/ week. | 618 patient consultations | General practice pharmacists were effective in making recommendations to improve patients’ pharmacotherapy and conducting activities to support patient education and disease state management. GPs accepted a high proportion of recommendations made by general practice pharmacists (88%). |
| Benson et al 2018 [51] | Conduct a preliminary process evaluation to inform the circumstances affecting implementation, intervention design and situational context of the integrated pharmacist intervention | Western Sydney, New South Wales | Mixed- method | 12: 5 | 3 months | Not described | 299 patient consultations  Interviews: 4 pharmacists and 5 GPs | Identified barriers and facilitators, and perspectives of stakeholders to standardise the model of inclusion of pharmacists into a general practice setting in a pilot study. |
| Deeks et al 2018 [52] | Describe the activities of general practice pharmacists | ACT | Quantitative non-RCT/ prospective observational study | 3: 3 | 6 months | 15.2–16 hours/ week | 3 pharmacists’ activities | Pharmacists were involved in the activities including medication reviews, patient and staff education, asthma care, smoking cessation, clinical audits, targeted deprescribing and post- hospitalisation medication reconciliation in general practices. |
| Deeks et al 2018 [53] | Evaluate the role of a general practice pharmacist in asthma management | ACT | Mixed-  method | 1: 1 | 13 months | 15.2–16 hours/ week | 136 patients | General practice pharmacists’ consultations improved asthma management in general practices. |
| Deeks et al 2018 [54] | Explore stakeholder perception about the role of the general practice pharmacist and the benefits, enablers, and barriers to implementation | ACT | Mixed- method | 3: 3 | 12 months | 15.2–16 hours/ week | Survey: 86 (44 patients and 42 practice staff)  Interviews: 20 | Patients, GPs, and other primary healthcare employees were supportive of pharmacists in general practice. |
| Peterson et al 2018 [55] | Illustrate the potential benefits of having a general practice pharmacist to facilitate deprescribing in older individuals | ACT | Qualitative/ case vignettes | 1: 1 | 12 months | 16 hours/ week* | 4 case vignettes | The general practice pharmacist improved medication safety and economic benefits may be derived by deprescribing in older persons. |
| Sake et al 2018 [61] | Elicit GPs’ perceptions about an integrated model for insomnia management with pharmacists in general practice | Greater Sydney, New South Wales | Qualitative/ thematic framework | N/A | N/A | N/A | 24 GPs from 14 general practices | Identified GPs’ views on the barriers, facilitators, service frameworks (service elements, service configuration, practice flow, shared medical record access, systems training), and performance indicators for insomnia management with pharmacists in general practice. |
| Mackie et al 2019 [62] | Explain the importance of the role of general practice pharmacist to manage medications in HIV patients (high HIV caseload general practices) | Melbourne | Qualitative/ Case study | Not described | 24 months | Not described | One case study | This case demonstrated that review of medications by  a specialist pharmacist within the general  practice can improve patient outcomes and prevent serious long-term consequences. |
| Deeks et al 2019 [56] | Determine whether a pharmacist can provide effective smoking cessation services within general practice | ACT | Quantitative non-RCT/ retrospective analysis | 1: 1 | 11.5 months | 15.2 hours/ week | 66 patients | General practice pharmacist provided an effective smoking cessation intervention in a general practice setting. |
| Baker et al 2019 [57] | Explore the perceptions of Australian accredited pharmacists and pharmacists working in general practices about current roles, facilitators and barriers, and remuneration expectations of practice pharmacists | Australia-wide | Mixed- method | N/A | N/A | N/A | Survey: Accredited pharmacists (n=65)  Interviews: pharmacists (n=20) | Identified current and future roles of general practice pharmacists, training requirements, remuneration expectations, current working relationships, and main barriers for the inclusion of general practice pharmacists. |
| Qazi et al 2020 [58] | Elicit pharmacists’ views and recommendations about models of collaboration in primary care involving the co-location of pharmacists within general practices to support GPs in providing asthma management services | Greater Sydney, New South Wales | Qualitative/ thematic analysis | N/A | N/A | N/A | 25 community pharmacists | Identified perspectives of community pharmacists on asthma management by general practice pharmacists; and key barriers and facilitators to general practice pharmacist-GP asthma care model. |
| Qazi et al 2020 [59] | Elicit GPs’ views and recommendations about  the inclusion of general practice pharmacists for supporting GPs in  providing asthma management services | Sydney and regional areas in New South Wales; and Melbourne (Victoria) | Qualitative/ thematic analysis | N/A | N/A | N/A | 23 GPs (participants n=9 had been working with a general practice pharmacist) | GPs in the study supported including a general practice pharmacist in primary care to manage asthma. GPs believed that general practice pharmacists can potentially improve the adherence to up-to-date asthma guidelines and deliver a broad range of asthma services in the evidence-based asthma management strategies. |
| Kosari et al 2020 [60] | Assess  the value-cost proposition of employing a pharmacist in a general  practice by calculating the cost of employing a pharmacist against the income generated,  through activities by general practice pharmacist (government-funded claimable activities, hours saved for the GPs, and potential payment from patients for pharmacist consultations) | ACT | Quantitative non-RCT/ prospective observational study | 2:2 | 19 weeks | 16 hours/week | 2 general practice pharmacists’ activities | Pharmacists’ activities in this study saved time for GPs allowing additional time for GP-patient consultations, and generated income to the general practice. |

N/A- Not Applicable, GP- General Practitioner, non-RCT- non-Randomised Control Trials, ACT-Australian Capital Territory

*Not described in the original article; additional information provided by the original investigator.
