# Supplementary material for: General practice pharmacists in Australia: A systematic review
Source: PLoS One. 2021 Oct 14;16(10):e0258674. doi: 10.1371/journal.pone.0258674 (PMC8516208; doi:10.1371/journal.pone.0258674)
Supplement: S3 Table — (DOCX) [file pone.0258674.s004.docx]

**S3 Table: Quality appraisal of the articles included in the review**

| **Qualitative studies (n= 10)** | | | | | | |
| --- | --- | --- | --- | --- | --- | --- |
| Study | Criteria | | | | | |
|  | 1.1. Is the qualitative approach appropriate to answer the research question? | 1.2. Are the qualitative data collection methods adequate to address the research question? | 1.3. Are the findings adequately derived from the data? | 1.4. Is the interpretation of results sufficiently substantiated by data? | 1.5. Is there coherence between qualitative data sources, collection, analysis and interpretation? | Sum |
| Freeman et al 2012 [40] | Yes | Yes | Yes | No | Yes | 4 |
| Freeman et al 2012 [42] | Yes | Yes | Yes | No | Yes | 4 |
| Tan et al 2013 [47] | Yes | Yes | Yes | Yes | Yes | 5 |
| Tan et al 2014 [45] | Yes | Yes | Yes | Yes | Yes | 5 |
| Bajorek et al 2015 [48] | Yes | Yes | Yes | Yes | Yes | 5 |
| Peterson et al 2018 [55] | Yes | No | No | Yes | Yes | 3 |
| Sake et al 2018 [61] | Yes | Yes | Yes | Yes | Yes | 5 |
| Mackie et al 2019 [62] | Yes | No | Yes | Yes | Yes | 4 |
| Qazi et al 2020 [58] | Yes | Yes | Yes | No | No | 3 |
| Qazi et al 2020 [59] | Yes | Yes | Yes | Yes | Yes | 5 |
| **Quantitative non-randomized control trials (n=8)** | | | | | | |
| Study | Criteria | | | | | |
|  | 3.1. Are the participants representative of the target population? | 3.2. Are measurements appropriate regarding both the outcome and intervention (or exposure)? | 3.3. Are there complete outcome data? | 3.4. Are the confounders accounted for in the design and analysis? | 3.5. During the study period, is the intervention administered (or exposure occurred) as intended? | Sum |
| Freeman et al 2012 [39] | No | Yes | Yes | No | Yes | 3 |
| Freeman et al 2013 [43] | No | Yes | No | No | Yes | 2 |
| Tan et al 2014 [46] | No | Yes | Yes | Yes | Yes | 4 |
| Benson et al 2018 [49] | Yes | Yes | Yes | No | Yes | 4 |
| Benson et al 2018 [50] | Yes | Yes | Yes | No | Yes | 4 |
| Deeks et al 2018 [52] | No | Yes | Yes | No | Yes | 3 |
| Deeks et al 2019 [56] | No | Yes | Yes | No | Yes | 3 |
| Kosari et al 2020 [60] | No | Yes | Yes | No | Yes | 3 |
| **Quantitative descriptive studies (n=2)** | | | | | | |
| Study | Criteria | | | | | |
|  | 4.1. Is the sampling strategy relevant to address the research question? | 4.2. Is the sample representative of the target population? | 4.3. Are the measurements appropriate? | 4.4. Is the risk of nonresponse bias low? | 4.5. Is the statistical analysis appropriate to answer the research question? | Sum |
| Freeman et al 2012 [41] | Yes | No | Yes | No | Yes | 3 |
| Freeman et al 2014 [32] | Yes | No | Yes | Yes | Yes | 4 |
| **Mixed-method studies (n=5)** | | | | | | |
| Study | Criteria | | | | | |
|  | 5.1. Is there an adequate rationale for using a mixed-method design to address the research question? | 5.2. Are the different components of the study effectively integrated to answer the research question? | 5.3. Are the outputs of the integration of qualitative and quantitative components adequately interpreted? | 5.4. Are divergences and inconsistencies between quantitative and qualitative results adequately addressed? | 5.5. Do the different components of the study adhere to the quality criteria of each tradition of the methods involved? | Sum |
| Benson et al 2018 [51] | Yes | Yes | Yes | No | No | 3 |
| Deeks et al 2018 [53] | Yes | Yes | Yes | No | Yes | 4 |
| Deeks et al 2018 [54] | Yes | Yes | Yes | No | No | 3 |
| Baker et al 2019 [57] | Yes | Yes | Yes | No | No | 3 |
| Tan et al 2014 [44] | Yes | Yes | No | No | No | 2 |
